# Current Hospital Topics

**Published:** 1907-04-27

**Authors:** 


### CURRENT HOSPITAL TOPICS.

### The Hospital Chapel.

A century ago, every new hospital of importance had a chapel, which was regarded by our forbears and sires as an essential portion of every "God's House of Pity," as these institutions were then regarded. In the subsequent decades the evolution of the hospital chapel has in some cases resulted in its extinction; in others it has led to the wise modification of associating the chaplaincy with the vicar of the parish in which the hospital is situated; whilst in others, again, the chapel has become the Valhalla of the heroes and devoted workers who have added to the reputation of the hospital, or whose lives and example have influenced others for good. The chapel at the *Dreadnought* Seamen's Hospital, Greenwich, is becoming a most interesting feature in modern London hospital life. It contains memorial windows recording the services of members of the medical staff and lay officials. It is endeared to the hearts of all who have been associated with the institution by many memories and special services of commemoration and worship, the latest of which has a special interest for hospital officials throughout the metropolis. It is pleasant to note the arrangements of this chapel, with its altar window illustrating the Parable of the Good Samaritan, together with the various gifts which beautify the altar and add a special interest to the chapel itself. The mere size of the staff of a modern hospital renders the chapel an important feature in its administration, and we should welcome a description, with illustrations, of every such chapel which has endeared itself to any hospital worker.

### Memorial to the Late Mr. Walter Adams.

On Sunday, April 13, Mr. Percival A. Nairne, chairman of the *Dreadnought* Seamen's Hospital, unveiled a memorial tablet in the chapel of that institution. When Mr. Nairne drew aside the Union Jack which covered the oak tablet, it was seen to bear the following inscription:—

> Walter Adams, Assistant Secretary. Born September 28, 1850, died March 6, 1906. To commemorate his service of thirty-five years in the Seamen's Hospital, his fellow-workers have raised a fund to bear his name and to augment the benefits of the Samaritan Fund, which he managed, with so deep and thoughtful an interest, in the cause of sick and needy seamen.

Mr. Nairne, who had known him for thirty years, testified to Mr. Adams's gentle disposition, his helpfulness to his seniors and to all who came in contact with him as a centre of information, his loving devotion to the interests of every convalescent patient who left the hospital. Medical officers, surgeons, nurses, and servants, all received courteous and kindly help from him, and he left a fragrant memory and an example stimulating all who were connected with the hospital. The Rev. S. Udny, Vicar of West Misted, Hampshire, gave an address, based upon the words, "Go thou and do likewise." In that chapel this gospel was always before their eyes, with the Master's words, "Take care of him." It was characteristic of Mr. Adams that he never lost faith, however bad a case might seem to be, for he had the gift of being able to put himself in another man's place, and to do for him what he would have had done for himself. He not only served the hospital, but Him in Whose name all hospitals must work. Without one word to help himself, Mr. Adams helped others by his deeds, and patiently bore the burden of those who were down in the world. He thus fulfilled the law of Christ. The whole service was impressive; the hymns were well chosen, and their memory will lovingly linger in the hearts of those who were present.

### Hospitals as Colleges of Hygiene.

In the report of the Stepney Visitors' Association, Dr. S. B. Atkinson suggests that among the work expected from our hospitals should be that of instructing those who attend them in the principles of health. Dr. Atkinson points out that the etiquette of the profession often hinders private medical men from giving demonstrations in hygiene in their own neighbourhood, and no one would wish the professional discouragement of what is often an insidious form of self-advertisement to be lessened. But the officials of our hospitals are in a different position from private practitioners; they are free from the suspicion of self-seeking, if, in addition to treating and nursing their patients, they give them some instruction in the laws of health. In-patients, and especially convalescents, whose energies are beginning to return, and who are apt to find time hang upon their hands, might be easily reached; but out-patients, who often need advice as to the management of their home life as much as the medicine for which they come to the hospital, would need to be dealt with by accredited health visitors, who would follow them up and give friendly advice and information. Working men and their wives might be gathered at meetings convened under the auspices of a religious or philanthropic association, and school children taught hygiene as part of their regular course of study. But the lecturers and the visitors would be accredited from the hospital, and should in fact be regarded as part of its staff. They would require to be paid from its funds; and as this is an entirely new departure, it is probable that the suggestion would require to come in the first place from subscribers or donors of a considerable amount. If the great hospital funds were to give the scheme their support, there would be little difficulty in carrying it through; but perhaps this could hardly be expected until it had been tried, experimentally, and proved useful. If successful, however, there is no doubt that the scheme which Dr. Atkinson outlines would add to the efficiency of hospitals by making them as powerful for the prevention as they are now for the relief of disease.

